# Australian arm of the International Spinal Cord Injury (Aus-InSCI) Community Survey: 2. Understanding the lived experience in people with spinal cord injury

**DOI:** 10.1038/s41393-022-00817-7

**Published:** 2022-06-15

**Authors:** James W. Middleton, Mohit Arora, Annette Kifley, Jillian Clark, Samantha J. Borg, Yvonne Tran, Sridhar Atresh, Jasbeer Kaur, Sachin Shetty, Andrew Nunn, Ruth Marshall, Timothy Geraghty

**Affiliations:** 1 John Walsh Centre for Rehabilitation Research, The Kolling Institute, Northern Sydney Local Health District, St Leonards, NSW Australia; 2 Sydney Medical School - Northern, Faculty of Medicine and Health, The University of Sydney, Sydney, NSW Australia; 3 State Spinal Cord Injury Service, Agency for Clinical Innovation, St Leonards, NSW Australia; 4 Spinal Outreach Service, Royal Rehab, Ryde, NSW Australia; 5 Faculty of Health and Medical Sciences, University of Adelaide, Adelaide, SA Australia; 6 Queensland Spinal Cord Injuries Service, Division of Rehabilitation, Princess Alexandra Hospital, Brisbane, QLD Australia; 7 The Hopkins Centre, School of Health Sciences and Social Work, Griffith University, Brisbane, QLD Australia; 8 Faculty of Medicine, Health and Human Sciences, Macquarie University, Sydney, NSW Australia; 9 Royal North Shore Hospital, Northern Sydney Local Health District, St Leonards, NSW Australia; 10 Prince of Wales Hospital, South Eastern Sydney Local Health District, Randwick, NSW Australia; 11 Victorian Spinal Cord Service, Austin Health, Heidelberg, VIC Australia; 12 South Australian Spinal Cord Injury Service, Central Adelaide Local Health Network, Adelaide, SA Australia

**Keywords:** Rehabilitation, Spinal cord diseases, Quality of life

## Abstract

**Study design:**

Cross-sectional survey.

**Objectives:**

To identify common problems across key domains of functioning, health and wellbeing, as well as evaluate self-reported quality of life (QoL) by people with SCI, examining differences by age, gender, injury characteristics and level of mobility.

**Setting:**

Data from four state-wide SCI clinical services, one government insurance agency and three not-for-profit consumer organisations.

**Methods:**

Participants were 18 years or over with SCI and at least 12 months post-injury, recruited between Mar’18 and Jan’19. The Aus-InSCI questionnaire comprised 193 questions, including socio-demographics, SCI characteristics, body functions and structures, activities and participation, environmental and personal factors, and appraisal of health and well-being. General linear model was used to examine differences in functioning and QoL.

**Results:**

Participants (mean age 57 years, range 19–94 years) with tetraplegia and/or complete injuries had more health problems, activity/participation problems and environmental barriers. However, self-rated overall QoL did not differ for injury level or completeness. Participants with more recent injuries exhibited lower independence levels, more mental health problems and poorer satisfaction with self and their living conditions. Major activity/participation problems related to intimate relationships and accessing public transportation. Less than half of the working age population were engaged in paid work. The top two environmental barriers frequently related to accessing public places or homes and unfavourable climatic conditions.

**Conclusions:**

This large, comprehensive community survey draws a detailed picture of the lived experience of people with SCI in Australia, identifying priority needs, gaps in services and barriers to achieving a full and satisfying life.

## Introduction

Spinal cord injury (SCI) has far-reaching physical, psychosocial and economic effects, impacting on the person injured, people close to them and society more broadly [1]. SCI may be associated with comorbidities, such as traumatic brain injury, psychological disorders and cognitive impairment [2], as well as secondary health conditions affecting multiple body systems, increasing lifetime risks of complications and rehospitalisation [3, 4]. Common secondary health conditions include urinary tract infections, spasticity, pressure injuries, pneumonia, autonomic dysreflexia, hypotension and pain [4, 5], which may impact negatively on productivity/employment, social participation and quality of life (QoL) [6]. In addition, ageing in a person with SCI may contribute to premature decline in health status, functioning and independence, with associated increase in health system utilisation [7]. This issue has gained greater prominence with improved life expectancy in many people with SCI approaching that of the general population [8].

Injury severity, functional limitations and health status following SCI alone do not determine the lived experience of disability in individuals with SCI [9]. A range of personal and environmental contextual factors may interact dynamically with the impairment to determine adjustment, participation and QoL outcomes following SCI. These include psychological attributes (e.g., personality, self-efficacy beliefs, appraisal and coping styles, motivation), financial resources, transportation, accessibility, legislative frameworks, employment opportunities, social support networks and societal views on SCI and disability [2, 10]. Additionally, the value of relationships and support provided by important people in the life of the person with SCI—such as partners, family members and friends—for re-establishing a sense of self-worth and affirmation of value for others that their injury had not changed is recognised [11].

Over the last decade, attention has been focused on promoting full integration of persons with disabilities in societies. The United Nations Convention on the Rights of Persons with Disabilities sets an agenda to achieve full and effective participation and inclusion in society for persons with disabilities [12], acknowledging that many different known and unknown barriers exist, including discriminating attitudes, lack of information or inaccessible environments. In a broad consultation process undertaken in Australia to develop a National Disability Strategy, lack of social inclusion and the multiple barriers to meaningful community participation faced by people with disabilities were issues frequently raised [13].

In a major step forward, the World Health Organisation (WHO) Global Disability Action Plan (GDAP) 2014–2021 (WHO, 2015) called on Member States to: (i) remove barriers and improve access to health services/programmes; (ii) strengthen and extend rehabilitation, assistive devices and support services, and community-based rehabilitation; and (iii) enhance collection of internationally comparable data on disability, and research on disability and related services [14]. The 2013 International Perspectives on Spinal Cord Injury (IPSCI) report developed by WHO and International Spinal Cord Society [1] echoed these recommendations, emphasising a need for better data collection to identify the most important problems and needs of people with SCI along the continuum of care and across the life span, learning from good practice models within and between countries.

This second paper in the series will provide an overview of the Australian arm of the International Spinal Cord Injury (Aus-InSCI) Community Survey data, with detailed person-centred information about the lived experience of SCI, capturing aspects of functioning and disability that are relevant to individuals with SCI, using a comprehensive International Classification of Functioning, Disability and Health (ICF)-based approach [15]. Ultimately, the goal of this research is to identify targets for improved clinical practice, health systems management, advocacy and policy change (in relation to evidence and rights, regulation, funding and supports) for people with SCI to be able to realise their potential, with choice and control about their own future [16].

The specific aims are to:

1. identify problems in functioning that are most frequently reported by people with SCI, including ICF domains of body function, activity, participation, environmental and personal factors,
2. examine differences in functioning by age, gender, injury characteristics and level of mobility,
3. evaluate self-reported QoL among people with SCI, including overall QoL and satisfaction with specific aspects of life, and
4. examine differences in QoL by age, gender, injury characteristics and level of mobility.

## Methods

### Study design and procedure

Study design, data linkage processes and methodology for Aus-InSCI (Middleton JW et al., Australian arm of the International Spinal Cord Injury (Aus-InSCI) Community Survey: 1. Population-based design, methodology and cohort profile, under review) and InSCI [17] have been described previously. In brief, Aus-InSCI forms part of the global cross-sectional InSCI study involving 22 countries and investigating the lived experience of people with SCI. Australian residents aged 18 years or older living in the community with a traumatic or non-traumatic SCI of at least 12-months duration post-injury participated. Participants were recruited and surveys completed between March 2018 and January 2019. The Aus-InSCI study combined data from nine different data custodians (hospital-based state-wide specialised SCI clinical services/units, not-for-profit community organisations and one government insurance agency) across four of the five Australian states (New South Wales, Queensland, South Australia and Victoria) with specialised spinal cord injury services. Eligible individuals were invited to participate by their respective data custodian and could complete survey as a paper-copy or online via enclosed unique participant login details.

### Data measures

The Aus-InSCI survey questionnaire comprises an international and national module with a total of 193 self-reported questions. The international module was developed by researchers at the Swiss Paraplegic Research Centre in conjunction with international SCI experts and included 125 questions related to ICF body functions and structures, activities and participation, environmental factors, personal factors, health and well-being. Items related to health system and economic resources of countries, including community rehabilitation, return-to-work programs, insurance schemes, and social welfare systems were also included [17]. Some key validated measures included Spinal Cord Independence Measure [18], Nottwil Environmental Factors Inventory Short Form [19], Spinal Cord Injury – Secondary Conditions Scale [20], the SF-36 vitality and mental health domains [21] and WHOQOL-BREF quality of life [22].

A further 68 questions relevant to Australian context were included in a national module, which was developed by a team of Australian stakeholders including consumers with SCI, insurers, researchers and clinicians working in the field of SCI. Included items were additional socio-demographic details (e.g., Australian state of residence, rural versus metropolitan location, Aboriginal or Torres Strait Islander origin), satisfaction with health service providers, as well as additional questions related to other factors impacting functioning (pain, fatigue, and skin problems), levels of physical activity, social integration, social injustice and quality of sleep. Some key validated measures included Injustice Experience Questionnaire [23], Fatigue Severity Scale [24], and Pittsburgh Sleep Quality Index [25]. A detailed description of the data collection items is provided in Appendix A and the Aus-InSCI survey questionnaire (national module) can be accessed as Appendix B.

### Data analysis

Statistical analyses were performed in SAS 9.4 (SAS Institute, Cary, North Carolina, USA) and R 3.6.1 (R Foundation for Statistical Computing). Demographic and injury characteristics and domains of lived experience of the Aus-InSCI population were described using percentages and means with 95% CI, median and interquartile range. Ratings of health problem severity, activity/participation problem severity, and difficulties caused by environmental barriers to participation were visualised using stacked bar charts. Average QoL outcomes, modified Spinal Cord Independence Measure-Self Report (m-SCIM-SR) scores, number of moderate to extreme activity/participation problems, Nottwil Environmental Factors Inventory – Short Form (NEFI-S) scores, number of reported health conditions, 36-item Short Form Survey (SF-36) mental health and vitality scores were compared between subgroups for demographic characteristics (age and sex) and injury characteristics (mobility category, lesion level and completeness, cause of injury and time since injury), both before adjustment using *t*-tests and non-parametric Kruskal Wallis tests, and after adjustment for other demographic and injury characteristics using the general linear model, which combines features of analysis of covariance and regression analysis.

## Results

### Aus-InSCI cohort characteristics

The Aus-InSCI survey involved 1579 participants, predominantly male (73%) with mean (SD) age 58 [14] years. Paraplegia (61%) was more common than tetraplegia, and incomplete lesions (67%) more common than complete. A traumatic cause of injury was recorded in 84%, most commonly related to transport (30%). Mean (SD) age at injury was 40 [18] years. Mean (SD) duration of injury was 17 [14] years. Cohort characteristics are presented in detail in the first paper of this series (Middleton JW et al., Australian arm of the International Spinal Cord Injury (Aus-InSCI) Community Survey: 1. Population-based design, methodology and cohort profile, under review).

### Function

Overall, most participants were able to achieve a level of independence in eating and drinking, washing, dressing, grooming and using the toilet, either on their own or through the use of assistive devices. Nonetheless, most still received day-to-day assistance (73%). The majority (85%) were able to sit without assistance, although of these one-quarter (26%) found this a moderate to extreme problem. Less than half (40%) were able to stand unassisted, and of these 64% found this a moderate to extreme problem. Based on ability to move moderate distances (10–100 m), 596 participants (41%) were ambulant with or without assistance, 516 (36%) used a self-propelled manual wheelchair, and 384 (27%) used an electric wheelchair or required assistance to operate a manual wheelchair.

### Activity/participation

**Fig. 1:**
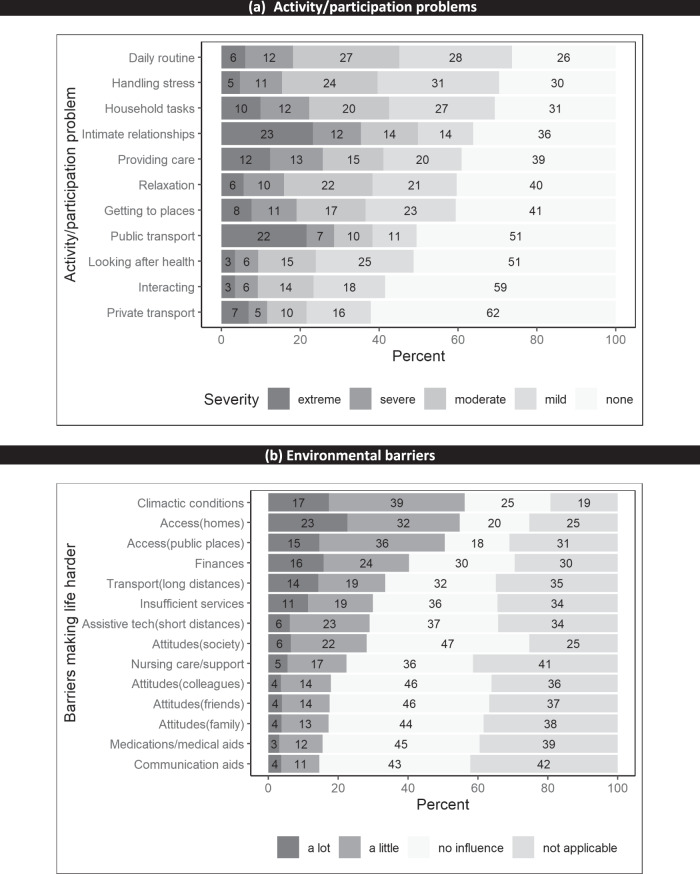
Activity/participation problems and environmental barriers.

Almost all participants (96%) had at least some activity/participation problems. The top five moderate to extreme problems involved intimate relationships (49%), carrying out daily routines (45%), getting household tasks done (43%), using hands and fingers (41%) and providing care or support for others (41%) (see Fig. 1a). The mean (SD) number of moderate to extreme problems was 4 (3.3). The most common severe to extreme problem was intimate relationships (35%).

### Work participation

Eighty-four percent of participants had a job before their injury. The absolute post-injury employment rate was 49.9%. For participants of working-age (18–65 years, *n* = 1078), 39% were currently in paid work at the time of filling the survey and 50% were receiving the disability pension. The predominant reason for not currently working related to health conditions or disability itself (74%). Pre-injury employment and vocational rehabilitation were key factors leading to higher employment rates [26]. Mean (SD) duration to return to work following SCI was 28 (36) months. Aus-InSCI employment outcomes are presented in detail elsewhere [26].

### Environmental barriers to participation

The mean (SD) NEFI-S total score was 34 [22]. The top five environmental barriers that made life a lot harder for participants were accessing public places or homes (15–23%), unfavourable climatic conditions (17%), problematic financial situation (16%), lack of or inadequate means of transportation over long distances (14%) and lack of or insufficient state services (11%) (see Fig. 1b).

### Secondary health conditions

**Fig. 2:**
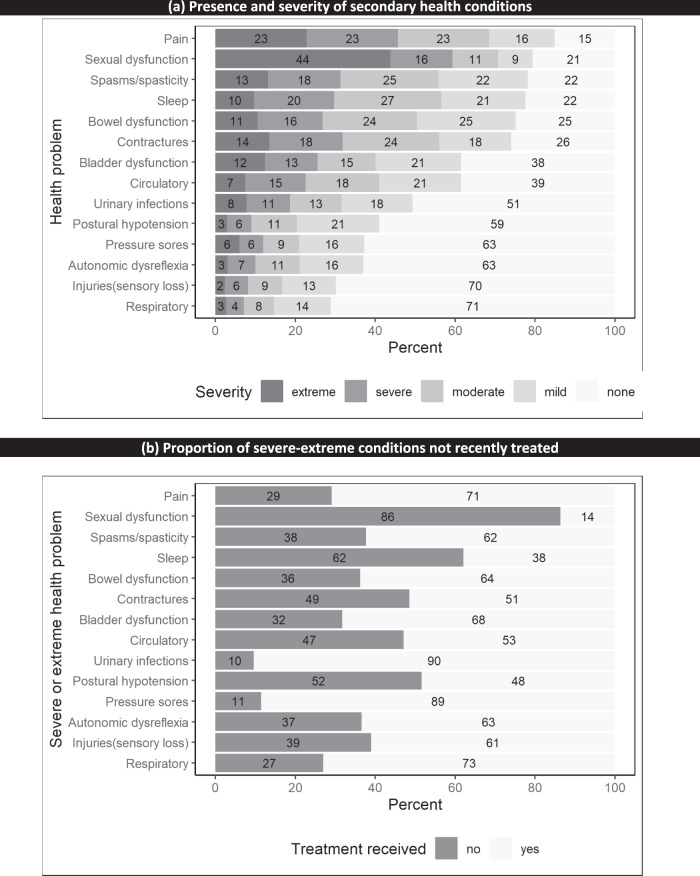
Self-reported secondary health conditions, severity and treatment outcomes.

Typically, participants reported experiencing eight health problems of any severity, three of which were rated as severe to extreme problems. The top five recent secondary health problems, reported by most participants, were pain (85%), sexual dysfunction (79%), muscle spasms or spasticity (78%), sleep problems (78%) and bowel problems (75%) (see Fig. 2a). Severe to extreme secondary health conditions most commonly involved sexual dysfunction (60%), pain (46%), contractures (32%), muscle spasms or spasticity (31%), sleep problems (30%), bowel problems (27%), and bladder problems (25%). A notably high proportion of severe to extreme conditions did not receive treatment; in particular, sexual dysfunction (86%), sleep problems (62%), hypotension (52%), and contractures (49%) (see Fig. 2b).

### Differences in function, health, activity/participation and environmental barriers by age, gender, injury characteristics and mobility level

Functional independence on m-SCIM-SR diminished on average with age and was poorer for more recent injuries. Activity/participation problems were more frequent for participants requiring electric or assisted manual wheelchairs and tetraplegia. Environmental barriers to participation were a greater problem for participants who were non-ambulant, or had complete tetraplegia, and were less of a problem for the oldest age groups. Males reported fewer activity/participation problems and environmental barriers than females (see Table 1). Participants who were non-ambulant, had complete tetraplegia, or participants aged between 31 and 60 years had more secondary health problems on average (see Table 1).

**Table 1:** Means, adjusted mean differences (md, 95% CI) and adjusted p values for differences by age, gender and injury characteristics in m-SCIM-SR total score for functional independence, number of moderate to extreme activity/participation problems, NEFI-S score for environmental barriers to participation and number of health conditions.

	m-SCIM-SR total score		No. of activity/participation problems		NEFI-S score		No. of health conditions	
	(range 0–100)		(moderate–extreme)		(range 0–100)		(mild–extreme)	
	mean	md (95% CI)	mean	md (95% CI)	mean	md (95% CI)	mean	md (95% CI)
Mobility category								
Ambulant	82	ref	3.7	ref	24.7	ref	7.4	ref
Motorised or assisted WC	29	−48 (−52, −45)	5	1.4 (0.9, 2.0)***	43.6	19 (15, 23)***	9.1	1.6 (1.1, 2.2)***
Self-operated manual WC	63	−20 (−23, −17)	3.4	0.02 (−0.5, 0.5)	36.7	11 (8, 15)***	8.1	0.7 (0.2, 1.2)**
* p* value		*–*		<0.0001		<0.0001		<0.0001
Injury level and extent								
Complete tetraplegia	27	ref	4.5	ref	42.3	ref	9.1	ref
Complete paraplegia	60	35 (29, 41)***	3.7	−1.0 (−1.7, −0.3)**	36.9	−5.1 (−9.5, −0.6)*	8.2	−0.9 (−1.6, −0.3)**
Incomplete tetraplegia	57	32 (26, 38)***	4.3	−0.4 (−1.1, 0.3)	33	−9.0 (−14, −4.6)***	8.4	−0.6 (−1.2, 0.04)
Incomplete paraplegia	73	51 (45, 57)***	3.8	−1.1 (−1.8, −0.4)**	31.7	−11 (−16, −6)***	7.7	−1.4 (−2.0, −0.7)***
* p* value		<0.0001		0.0003		<0.0001		<0.0001
Cause of injury								
Non-traumatic	63	ref	4.4	ref	34.7	ref	7.9	ref
Traumatic	61	−2.7 (−7.2, 1.8)	3.8	−0.5 (−1.0, −0.02)*	33.4	−1.9 (−5.1, 1.3)	8.1	−0.09 (−0.6, 0.4)
* p* value		0.2		0.04		0.2		0.7
Injury duration								
Up to 5 years	66	−2.7 (−5.8, 0.4)	4	0.4 (−0.2, 1.0)	31.7	3.0 (−0.4, 6.5)	7.7	−0.14 (−0.66, 0.38)
6–15 years	61	−5.3 (−8.1, -2.6)***	3.9	0.3 (−0.2, 0.9)	34.7	3.9 (0.8, 6.9)*	8.2	0.08 (−0.4, 0.6)
16–25 years	62	−1.0 (−4.2, 2.3)	4.1	0.4 (−0.2, 1.0)	34.3	2.4 (−1.2, 6.0)	8.2	0.0 (−0.6, 0.6)
26+ years	59	ref	3.7	ref	32.7	ref	8.2	ref
* p* value		0.0005		0.4		0.09		0.8
Gender								
Female	59	ref	4.4	ref	36.1	ref	7.9	ref
Male	62	3.2 (0.9, 5.6)**	3.8	−0.5 (−0.9, −0.06)*	32.6	−3.3 (−5.9, −0.7)*	8.1	0.2 (−0.2, 0.6)
* p* value		0.006		0.02		0.01		0.3
Age group								
18–30 years	58	11 (5.2, 18)***	3.2	−0.6 (−1.7, 0.5)	38.5	11 (4, 18)**	7.3	−0.06 (−1.1, 1.0)
31–45 years	64	10 (5.6, 15)***	4.1	0.5 (−0.3, 1.4)	36.4	12 (6, 18)***	8.4	1.0 (0.1, 1.9)*
46–60 years	62	7.4 (3.0, 12)***	4.2	0.8 (0.05, 1.6)*	35.7	13 (8, 18)***	8.4	1.1 (0.3, 1.9)**
61–75 years	61	5.7 (1.4, 10)***	3.7	0.2 (−0.6, 1.0)	31.8	8 (3, 13)***	7.8	0.4 (−0.4, 1.2)
76+ years	59	ref	3.7	ref	23.7	ref	7.4	ref
* p* value		<0.0001		0.003		<0.0001		0.0003

*CI* confidence interval, *md* mean difference (adjusted), *mod* moderate, *NEFI-S* Nottwil Environmental Factors Inventory Short Form, *m-SCIM-SR* modified version of the Spinal Cord Independence Measure for Self-Report, *ref* reference category.

The overall *p* values shown are from type-III F tests for any difference in mean outcome among categories of each characteristic.

*p < 0.05, **p < 0.01, ***p < 0.001 for individual difference against the reference categories.

*p* values for age are adjusted for sex, duration, cause, level and completeness and mobility category.

*p* values for sex are adjusted for age, duration, cause, level and completeness and mobility category.

*p* values for injury duration are adjusted for age, sex, cause, level and completeness and mobility category.

*p* values for cause of injury are adjusted for age, sex, duration.

*p* values for lesion level and completeness adjusted for age, sex, duration and cause.

*p* values for mobility category adjusted for age, sex, duration, cause, lesion level and completeness.

### Personal factors

Participants positively rated their sense of autonomy (74%), ability to maintain important relationships (75%), and sense of belonging (64%). Participants were less certain that they would be able to maintain their health (50%), and most were worried about their future (60%). Only a minority believed they would be able to fulfil their hopes and dreams (31%).

### Perceived social injustice, social support and integration

Sixty per cent of participants frequently or always felt that people did not understand the severity of their condition and roughly half frequently or always felt they just wanted their life back. Most participants positively rated the respect they received from others (80%) and received help and support from people close to them when they needed it (86%). However, roughly one-quarter (23%) felt they had very little chance to show their capabilities fully.

### Mental health and vitality

Most participants (70%) reported some problem with fatigue. Feeling persistently tired (35%) or worn out (26%) was more common than feeling persistently nervous (9%), down in the dumps (8%) or depressed (12%).

### Differences in mental health, vitality and psychosocial factors by age, gender, injury characteristics and mobility level

Mental health scores were poorer on average among participants with non-traumatic or recent injuries, and were higher in the oldest age groups. SF-36 vitality scores were higher on average among participants using self-propelled manual wheelchairs, with traumatic injuries or in the oldest age groups, and poorer on average for participants with incomplete tetraplegia. Self-efficacy scores were higher on average among participants with traumatic injuries and manual wheelchair users. Social integration scores were better in the oldest age groups, as well as in participants with more than 25 years post their SCI (see Table 2).

**Table 2:** Means, adjusted mean differences (md, 95% CI) and adjusted p values for differences by age, gender and injury characteristics in SF-36 scores for mental health and vitality, self-efficacy score and social integration score.

	Mental health score		Vitality score		Self-efficacy score		Social integration score	
	(norms-based score)		(norms-based score)		(range 0–100)		(range 0–100)	
	Mean	md (95% CI)	Mean	md (95% CI)	Mean	md (95% CI)	Mean	md (95% CI)
Mobility category								
Ambulant	45.1	ref	41	ref	66.1	ref	69.2	ref
Motorised or assisted WC	44.6	−1.7 (−3.6, 0.1)	41.4	−0.08 (−1.8, 1.7)	65.1	−3.7 (−6.9, −0.4)*	68.1	−2.0 (−4.5, 0.5)
Self-operated manual WC	46.8	1.3 (−0.5, 3.1)	44.2	2.8 (1.1, 4.5)**	69.4	2 (−1.2, 5.2)	70	0.6 (−1.8, 3.1)
*p* value		0.003		0.0005		0.002		0.08
Injury level and extent								
Complete tetraplegia	47.5	ref	44	ref	70.1	ref	70.5	ref
Complete paraplegia	46.4	−0.8 (−3.3, 1.7)	43.6	0.05 (−2.3, 2.4)	67.6	−1.7 (−6.2, 2.8)	69.6	−0.4 (−3.8, 3.0)
Incomplete tetraplegia	44.7	−2.2 (−4.6, 0.3)	40.6	−3.0 (−5.3, −0.7)*	67.4	−1.5 (−5.9, 2.9)	69.2	−0.8 (−4.1, 2.6)
Incomplete paraplegia	44.9	−1.8 (−4.3, 0.7)	42	−0.9 (−3.3, 1.5)	65.6	−1.7 (−6.2, 2.7)	68.2	−1.3 (−4.7, 2.1)
*p* value		0.2		0.0008		0.9		0.8
Cause of injury								
Non-traumatic	43.7	ref	39.3	ref	61.7	ref	67.5	ref
Traumatic	45.8	1.9 (0.1, 3.6)*	42.7	3.1 (1.5, 4.8)***	67.8	6.3 (3.1, 9.4)***	69.5	2.5 (0.1, 4.9)*
* p* value		0.03		0.0002		<0.0001		0.03
Injury duration								
Up to 5 years	44.2	−2.5 (−4.6, −0.5)*	41.4	−0.3 (−2.2, 1.6)	65.5	−2.4 (−5.9, 1.2)	68.6	−3.1 (−5.8, −0.4)*
6–15 years	45.2	−1.0 (−2.8, 0.8)	42	0.04 (−1.7, 1.8)	66.6	−1.7 (−4.9, 1.5)	68.8	−2.8 (−5.2, −0.3)*
16–25 years	45.5	−1.1 (−3.2, 1.0)	42.6	0.3 (−1.6, 2.3)	67.8	−0.7 (−4.4, 2.9)	67.6	−3.8 (−6.6, −1.0)**
26+ years	47.5	ref	43.3	ref	68.6	ref	71.8	ref
*p* value		0.09		0.9		0.6		0.03
Gender								
Female	44.4	ref	40.4	ref	65.4	ref	68	ref
Male	45.9	0.7 (−0.9, 2.2)	42.8	1.4 (0, 2.8)	67.4	0.5 (−2.2, 3.2)	69.6	0.6 (−1.5, 2.6)
*p* value		0.4		0.05		0.7		0.6
Age group								
18–30 years	45.2	−5.7 (−10, −1.1)**	42.6	−3.2 (−6.9, 0.6)	68.5	−4.2 (−12, 2.9)	70.8	−3.5 (−8.9, 1.8)
31–45 years	43.1	−8.0 (−11, −4.9)***	40.6	−5.1 (−7.9, −2.2)***	66.6	−7.2 (−13, −1.9)**	67.5	−7.0 (−11, −2.9)***
46–60 years	44.6	−6.1 (−8.9, −3.3)***	41.1	−4.1 (−6.6, −1.5)**	65.8	−7.5 (−13, −2.6)**	67.3	−8.0 (−12, −4.3)***
61–75 years	46.7	−3.5 (−6.3, −0.8)*	43.4	−1.2 (−3.7, 1.3)	67.5	−5.0 (−9.8, −0.2)*	70.8	−4.2 (−7.8, −0.6)*
76+ years	48.7	ref	43.7	ref	68.3	ref	71.7	ref
*p* value		<0.0001		<0.0001		0.03		<0.0001

*CI* confidence interval, *md* mean difference (adjusted).

The overall *p* values shown are from type-III F tests for any difference in mean outcome among categories of each characteristic.

*p < 0.05, **p < 0.01, ***p < 0.001 for individual difference against the reference categories.

*p* values for age are adjusted for sex, duration, cause of injury, level and completeness and mobility category.

*p* values for sex are adjusted for age, duration, cause of injury, level and completeness and mobility category.

*p* values for injury duration are adjusted for age, sex, cause of injury, level and completeness and mobility category.

*p* values for cause of injury are adjusted for age, sex, duration.

*p* values for lesion level and completeness adjusted for age, sex, duration and cause of injury.

*p* values for mobility category adjusted for age, sex, duration, cause of injury, lesion level and completeness.

### Self-rated QoL in the Aus-InSCI cohort

**Fig. 3:**
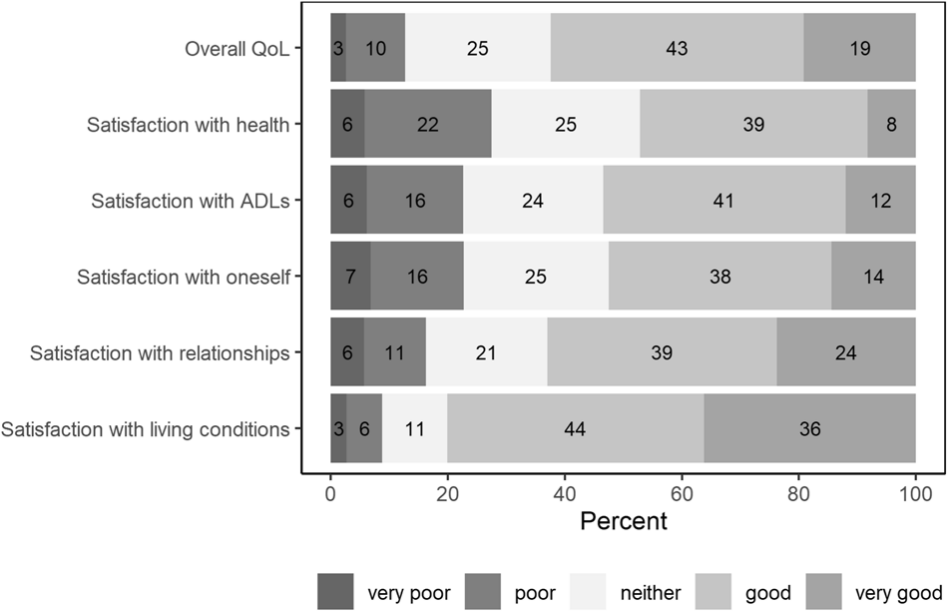
Self-reported overall quality of life (QoL) and satisfaction with health, activities of daily living (ADLs), oneself, relationships and living conditions.

Most participants rated overall QoL as good or very good (62%). Only 13% rated their overall QoL as poor or very poor; however, dissatisfaction with health, oneself, and the ability to perform activities of daily living were more common (22–28%) (see Fig. 3). Contributing factors to QoL are examined in detail in third paper of this series.

### Differences by age, gender, lesion characteristics and mobility level in self-rated QoL

In the adjusted analyses, overall QoL and health satisfaction were poorer on average among participants with non-traumatic rather than traumatic injuries, short injury duration (<5 years), in middle age ranges or using assisted manual or electric wheelchairs. These differences by age group reflected all subdomains of life quality, while differences by mobility category and cause of injury reflected all subdomains except satisfaction with relationships. Differences by injury duration primarily reflected differences in satisfaction with oneself and living conditions (see Table 3).

**Table 3:** Differences in quality of life and satisfaction ratings by age, sex, injury duration, injury characteristics and mobility category.

	Overall QOL (0-100)	Satisfaction with health (0-100)	Satisfaction with routine (0-100)	Satisfaction with oneself (0-100)	Satisfaction with relationships (0-100)	Satisfaction with living conditions (0-100)
Mean (95% CI)	66 (65, 68)	56 (54, 57)	59 (57, 61)	59 (57, 61)	66 (64, 68)	76 (74, 78)
Median (IQR)	75 (50, 75)	50 (25, 75)	75 (50, 75)	75 (50, 75)	75 (50, 75)	75 (75, 100)
Mobility category	mean (95% CI)	mean (95% CI)	mean (95% CI)	mean (95% CI)	mean (95% CI)	mean (95% CI)
Ambulant	67.4 (65.5, 69.3)^ref^	55.2 (53.0, 57.4)^ref^	60.2 (58.1, 62.4)^ref^	58.4 (56.1, 60.7)	66.3 (64.0, 58.6)	77.0 (75.0, 79.0)^ref^
Assisted or electric wheelchair	61.2 (58.3, 64.1)***	51.5 (48.6, 54.3)	50.0 (47.0, 53.0)***	57.0 (54.1, 59.9)	66.3 (63.4, 69.2)	72.9 (70.2, 75.6)**
Self-operated wheelchair	69.1 (67.1, 71.2)	58.8 (56.5, 61.0)*	64.5 (62.2, 66.7)	61.7 (59.2, 64.2)	66.1 (63.6, 68.6)	77.9 (75.9, 80.0)
*p* value	<0.0001	0.0004	<0.0001	0.04	0.8	0.0009
Lesion level and completeness	mean (95% CI)	mean (95% CI)	mean (95% CI)	mean (95% CI)	mean (95% CI)	mean (95% CI)
Complete tetraplegia	64.3 (59.8, 68.9)	54.5 (49.3, 59.7)	51.2 (45.8, 56.7)^ref^	59.4 (54.1, 64.8)	66.1 (60.7, 71.5)	78.3 (74.0, 82.5)
Complete paraplegia	66.5 (63.8, 69.2)	56.9 (54.2, 59.7)	62.6 (59.8, 65.3)***	61.8 (58.8, 64.9)	66.5 (63.5, 69.5)	76.1 (73.4, 78.7)
Incomplete tetraplegia	66.1 (63.7, 68.5)	54.4 (51.8, 57.0)	55.7 (53.0, 58.5)*	57.5 (54.8, 60.2)	65.4 (62.7, 68.2)	75.2 (72.7, 77.7)
Incomplete paraplegia	66.9 (64.8. 69.0)	54.9 (52.7, 57.2)	60.6 (58.3, 62.9)***	57.8 (55.4, 60.2)	66.1 (63.6, 68.5)	76.2 (74.1, 78.3)
*p* value	0.1	0.5	<0.0001	0.4	0.9	0.7
Cause of injury	mean (95% CI)	mean (95% CI)	mean (95% CI)	mean (95% CI)	mean (95% CI)	mean (95% CI)
Traumatic	67.3 (65.9, 68.7)	56.7 (55.2, 58.2)	60.0 (58.5, 61.5)	60.2 (58.6, 61.7)	66.4 (64.8, 68.0)	76.7 (75.3, 78.1)
Non-traumatic	62.9 (59.8, 65.9)	49.5 (46.1, 52.9)	54.7 (51.1, 58.3)	55.0 (51.5, 58.6)	65.4 (61.8, 69.1)	73.8 (70.5, 77.1)
*p* value	0.015	0.0003	0.02	0.02	0.14	0.01
Injury duration	mean (95% CI)	mean (95% CI)	mean (95% CI)	mean (95% CI)	mean (95% CI)	mean (95% CI)
Up to 5 years	62.8 (60.1, 65.6)***	54.0 (51.2, 56.7)	56.1 (53.1, 59.0)	55.1 (52.0, 58.2)**	64.9 (62.0, 67.9)	73.1 (70.3, 75.8)*
6–15 years	67.2 (65.0, 69.3)	55.8 (53.4, 58.2)	59.8 (57.4, 62.2)	59.6 (57.1, 62.1)	67.0 (64.5, 69.4)	78.6 (76.5, 80.6)
16–25 years	67.3 (64.3, 70.3)	55.6 (52.3, 58.8)	60.5 (57.2, 63.8)	59.5 (56.2, 62.9)	64.4 (60.8, 67.9)	73.4 (70.2, 76.7)
26+ years	69.4 (66.9, 71.9)^ref^	57.4 (54.7, 60.2)	60.4 (57.6, 63.2)	63.2 (60.4, 66.0)^ref^	68.3 (65.4, 71.2)	78.1 (75.7, 80.4)^ref^
p *value*	0.002	0.8	0.15	0.014	0.5	0.0004
Sex	mean (95% CI)	mean (95% CI)	mean (95% CI)	mean (95% CI)	mean (95% CI)	mean (95% CI)
Female	66.7 (64.3, 69.2)	52.7 (50.0, 55.4)	57.1 (54.3, 59.8)	55.8 (53.0, 58.6)	66.2 (63.4, 69.0)	75.6 (73.1, 78.1)
Male	66.5 (65.0, 68.0)	56.6 (55.0, 58.2)	59.9 (58.3, 61.5)	60.6 (59.0, 62.3)	66.2 (64.5, 67.9)	76.4 (75.0, 77.9)
*p* value	0.7	0.11	0.11	0.024	0.6	0.8
Age group	mean (95% CI)	mean (95% CI)	mean (95% CI)	mean (95% CI)	mean (95% CI)	mean (95% CI)
18–30 years	73.2 (68.0, 78.4)	62.7 (56.9, 68.5)	63.8 (57.5, 70.0)	64.5 (57.9, 71.1)	68.1 (61.9, 74.3)*	75.7 (70.2, 81.2)*
31–45 years	66.7 (63.6, 69.8)*	52.7 (49.2, 56.1)**	58.4 (54.9, 62.0)	54.5 (50.7, 58.2)***	60.6 (56.9, 64.3)***	71.9 (68.4, 75.3)***
46–60 years	65.2 (63.0, 67.4)**	53.3 (50.9, 55.6)***	57.5 (55.1, 59.9)	56.6 (54.1, 59.1)***	62.0 (59.3, 64.7)***	74.0 (71.8, 76.2)***
61–75 years	66.3 (64.1, 68.4)	57.1 (54.8, 59.3)	60.4 (58.1, 62.6)	61.4 (59.1, 63.7)**	70.3 (68.1, 72.6)*	78.7 (76.7, 80.7)**
76+ years	69.6 (65.7, 73.5)^ref^	59.9 (55.6, 64.2)^ref^	59.8 (55.3, 64.4)	67.6 (63.4, 71.7)^ref^	73.9 (69.9, 77.8)^ref^	82.0 (78.7, 85.3)^ref^
*p* value	0.001	<0.0001	0.007	<0.0001	<0.0001	<0.0001

*CI* confidence interval, *IQR* Interquartile range, *QoL* quality of life.

Outcomes were measured on five-point scales ranging from very poor to very good or very dissatisfied to very satisfied and were rescaled to range from 0 (worst) to 100 (best).

The overall *p* values shown are from type-III F tests for any difference in mean outcome among categories of each characteristic.

*p < 0.05, **p < 0.01, ***p < 0.001 for individual difference against the reference categories.

*p* values for age are adjusted for sex, duration, cause of injury, level and completeness and mobility category.

*p* values for sex are adjusted for age, duration, cause of injury, level and completeness and mobility category.

*p* values for injury duration are adjusted for age, sex, cause of injury, level and completeness and mobility category.

*p* values for cause of injury are adjusted for age, sex, duration.

*p* values for lesion level and completeness adjusted for age, sex, duration and cause.

*p* values for mobility category adjusted for age, sex, duration, cause, lesion level and completeness.

## Discussion

The Aus-InSCI study represents the largest and most wide-ranging community survey of health-related issues, functioning, social inclusion, economic participation, environmental factors and QoL in the SCI population that has ever been conducted in Australia, building for the first time a detailed picture of the lived experience of people with SCI in Australia. Several countries have previously undertaken large national surveys depicting major aspects in the lives of people with SCI and guiding policy makers and service providers to enhance QoL [27, 28]. However, being part of an international study, Aus-InSCI survey provides a unique opportunity for benchmarking Australia on different ICF domains in comparison to the other 21 participating countries, and also facilitates the learning of effective strategies and best practice models from different countries [16].

In our study, environmental barriers related to accessing public places or homes, unfavourable climatic conditions, financial difficulties, inadequate transportation, insufficient services and negative societal attitudes made life more difficult for many participants. Reduced self-care ability, worse mental health and low income have been shown to be strong predictors of environmental barriers at an individual level [29]. In Canada, Noreau et al (2014) also found that environmental barriers to services remain an issue that requires review of certain needs, for example, accessible housing, attendant care, transportation, and income support for people with SCI to support independent living in the community [30]. The study results also showed that severe to extreme activity/participation problems were common and varied, including difficulty with standing, accessing public transportation, using hands and fingers, providing care or support for others and getting household tasks done. Together these findings highlight that in addition to provision of physical assistance, individuals with SCI need to receive good psychosocial support, have accessible and affordable transportation options and appropriate assistive technologies and specialised equipment to achieve full integration into the community, in a way that empowers them with independence, choice and control. Ensuring new transport infrastructure is built to highest standards of accessibility for people with SCI, as well as finding common sense solutions, requires governments to follow inclusive planning processes guided by the social model of disability and understanding the whole-of-journey experience and interaction between modes of transport and the physical environment. The shift away from traditional funding programs to an individual funding model, now being delivered through new National Disability Insurance Scheme, provides more choice and flexibility to individuals but requires ongoing monitoring and evaluation. These schemes also offer better access to assistive technology and products that can reduce need for additional care and support services.

Previous research has shown that some aspects of participation and role functioning may be more restricted than others, including certain roles within the family, work and education [31]. In our study, less than half of the working age population were engaged in paid work, despite many feeling that they were unable to work. Returning to work or gaining new employment after SCI signifies positive community reintegration and leading a ‘normal life’ [32], although achieving this can often be very challenging. Work-related issues have been reported separately [26]. Increased access to vocational rehabilitation and support for persons with SCI during rehabilitation or afterwards when transitioning to work or study is key, as well as addressing modifiable factors or barriers to employment by ensuring access to education and training, return to driving programs and transportation, and managing health-related issues.

Most participants felt they had control over their lives and good support when needed, displaying a good deal of resilience in handling their situation, although a majority also perceived that other people did not really understand the severity of their condition and only one-third believed they could achieve their aspirations. While most were worried about their health and/or future, only 10-15% reported persistent problems with depressive mood or anxiety or felt dissatisfied with their overall life quality. Interpersonal interactions and intimate relationships are integral to the lived experience and our results strongly suggest there is a need for proactive relationship support and psychoeducation, beginning in rehabilitation and maintained during community integration. A recent scoping review identified various environmental or personal factors that may facilitate the development and maintenance of strong relationships after SCI, including partner and social support, reciprocity in relationships, and presenting oneself positively [33]. Conversely, factors impeding relationships were reported to include physical environmental barriers, real and perceived social biases, and poor self-image. Maximising autonomy and reducing the impact of physical dependence upon relationships may be accomplished by learning from peers with SCI, being informed, setting goals, planning and organisation, learning to be assertive, asking for and accepting help, and learning to deal with the reactions of others [33]. This points to a need for integrated person-centred care and peer support programs, with interventions involving social skills development, practical information to assist coping with the physical and psychological challenges, and ongoing psychological support for individuals living with SCI.

People with SCI overall are known to experience a high burden of secondary health problems [4]. Multimorbidity was common among the Australian cohort. For value-based healthcare delivery to improve health-related outcomes that matter to people with SCI, our results highlight a need to target management of debilitating pain, sexual dysfunction, spasms, sleep disorder, bowel problems and fatigue. These prevalent and often severe secondary health conditions significantly impact the lives of people with SCI in Australia, interfering with physical functioning and independence, mood, as well as with work, social and family life. These findings are consistent with recent research from Canada [28] and Switzerland [34], reporting chronic pain, sexual dysfunction, spasticity, musculoskeletal disorders, urinary tract infections and bowel problems occurring commonly (between 50% and 74%). Despite the frequency and severity of secondary health conditions, only some individuals reported current problems in accessing health care when needed, mostly due to financial issues or lack of/perceived inadequacy of available services. Previous research in Australia [35] and in Canada [30] has shown that limited SCI-specific knowledge of local service providers and treatment affordability are important barriers to health care needs being met. Low prevalence rates negatively impact the acquisition of necessary equipment and knowledge required to optimally care for patients with SCI in typical primary care settings. Practice constraints also result in episodic rather than preventive care [36]. Novel approaches are needed combining the use of integrated and shared care models supported via telehealth or other media, for improved access to expertise for decision-making around complex health issues, along with evidence-informed point-of-practice tools [37] and self-management tools [38, 39]. We have previously advocated use of the NSW Spinal Outreach Service Health Questionnaire (SOS-HQ) to support collaborative primary care and cue regular health screening for surveillance, early intervention and preventive care [37].

Disconcertingly, a large proportion of chronic problems reported in the Aus-InSCI survey were not recently treated, even when rated as severe or extreme, most notably for sexual dysfunction and sleep disorders. Consistent with previous research [34], sexual problems and needs unfortunately remain largely unaddressed in the community, with sexual dysfunction being rated not only as the most severe problem (44% severe or extreme) but also demonstrating the largest unmet need (being untreated in 86% of those with severe to extreme conditions), impacting adversely on self-identity, social participation, relationships and quality of life. Previous research has identified recovery of sexual function and bowel and bladder control as higher priorities than walking for most individuals with SCI [40], with over 80% of people with SCI indicating that improving their sexual function would improve their QoL. The primary motivating reason for pursuing sexual activity is a need for intimacy rather than fertility [41]. Sexuality remains a largely neglected area of rehabilitation yet continues to be one of the high priority areas for people with SCI. New initiatives are needed for design and delivery of an integrated, comprehensive multi-disciplinary approach, including education, peer-counselling, psychosexual therapy, assistive devices and other strategies to address all aspects of sexual intimacy and activity. Sleep disorder had the second largest unmet need (62% of severe and extreme conditions untreated) and also deserves more attention, given its major impact on health, function and quality of life [42]. Increasing consumer and clinician awareness about the negative impact of poor sleep quality on daily functioning, participation and quality of life is needed. Longitudinal studies are required to understand the complex relationships between sleep, pain, fatigue and mental health, and outcomes for people with SCI, in order to inform improved treatments.

Chronic pain remains a pervasive problem impacting negatively on activities, participation, mood and quality of life. The extent to which pain interferes with activity and participation may reflect a person’s overall coping ability and self-efficacy [43]. In the current study, despite most participants using three or more treatment strategies to manage their pain, effectiveness of treatment was rated at just over 5/10. This points to the need for widespread implementation of comprehensive, person-centred SCI pain self-management programs [44], as well as innovative treatment approaches, including targeting supraspinal mechanisms [45].

The Aus-InSCI survey has helped identify priorities of people with SCI in relation to their lived experience. Their priorities must, in turn drive deliberations on options for changes in policy, improvement in service delivery systems or funding, or establishment of ‘best-practice’ clinical intervention/s and care provision. Review of relevant legislation, standards and policy frameworks will help to identify targets at the levels of government policy-making (in relation to evidence and rights). A key target for service development is better integration across the continuum of care with improved care transitions from hospital into the community, as well as increased access to equipment, care and support services. Policy briefs containing policy options, outlining expected benefits, costs and relevant implementation paths, along with barriers and facilitators to the implementation of each option will be developed to guide stakeholder dialogues with key policy makers and healthcare planners at state and national levels, in relation to resource allocation, planning and delivery of future healthcare services for Australians with SCI. A pragmatic meta-theoretical framework, the Consolidated Framework for Implementation Research, will complement the study findings through synthesizing knowledge and generating an understanding about potential barriers/facilitators and what works in specific contexts to develop new context-specific strategies related to intervention, settings, individuals, and implementation process [46]. The engagement and participation of a broad range of research partners (from health services, government agencies and non-government SCI consumer organisations) provides a powerful vehicle for advocacy, dissemination, codesign and translation of the study’s findings into practice improvement and policy change, taking into account the local and national context. There is growing evidence from randomised controlled trials of the value of evidence-informed consumer engagement on achievement of relevant and positive outcomes of health policy, research and services, with embedded evaluation [47]. Further, partnering with peers to challenge social paradigms of disability and societal attitudes could be complemented by education and media campaigns to reduce discrimination and promote social inclusivity. A five-year follow-up survey planned for 2023 will add both longitudinal and cross-sectional data related to lived experience and a capacity to evaluate any short-term changes with implementation activities, nationally and internationally.

## Supplementary information

Appendix A: Description of Aus-InSCI data measures

Appendix B: Aus-InSCI National Module

## Data Availability

De-identified data are available upon request and with permission gained from the Aus-InSCI Community Survey National Scientific Committee.
