# Supplementary material for: Australian arm of the International Spinal Cord Injury (Aus-InSCI) Community Survey: 2. Understanding the lived experience in people with spinal cord injury
Source: Spinal Cord. 2022 Jun 15;60(12):1069–79. doi: 10.1038/s41393-022-00817-7 (PMC9712098; doi:10.1038/s41393-022-00817-7)
Supplement: Supplementary file 1 — Appendix A: Description of Aus-InSCI data measures [file 41393_2022_817_MOESM1_ESM.pdf]

Appendix A: Description of Aus-InSCI data measures

Outcome measure	Description
**A. International Module**	
**Sub-module #1: Sociodemographic characteristics**	*Number of items*: 10<br>*Description*: this included questions related to gender, age, country of birth, marital status, living status, assistance with day-to-day activities, education and training, household income and social status (using McArthur Scale of subjective social status).<br>*Interpretation*: each variable was treated as a standalone continuous, dichotomous and/or categorical variable (see details below):

Variables	Data type
Gender	Dichotomous (male/female)
Age	• Continuous<br>• Categorical (DeVivo classification)
Marital status	Categorical
Living status	Categorical
Assistance	• Dichotomous (Yes/No)<br>• Categorical
Education and training	• Continuous (total years)<br>• Categorical
Household income	• Continuous (income index)<br>• Categorical
Social ladder	Continuous (0 to 10), higher score better outcome

NB: The variable was used as continuous score ranging from 1-10, with higher values indicating higher subjective social status.

Outcome measure	Description
**Sub-module #2: Injury characteristics**	*Number of items*: 4<br>*Description*: this included questions related to level of injury, completeness of injury, cause of injury and date of injury.<br>*Interpretation*: each variable was treated as a standalone dichotomous or categorical variable (see details below):

Variables	Data type
Level of injury	Dichotomous (tetraplegia versus paraplegia)
Completeness of injury	Dichotomous (complete versus incomplete)
Cause of injury	• Dichotomous (traumatic versus non-traumatic)<br>• Categorical
Date of injury (as time post injury)	• Continuous<br>• Categorical (derived from year of birth, year of injury and time of interview)

Outcome measure	Description
**Sub-module #3: Energy and feelings**	*Number of items*: 9

	Description: The SF-36 vitality (VT) and mental health (MH) domains were used to assess energy levels and feelings of anxiety and/or depression over the last four weeks. Domain summary scores were obtained using norms-based scoring for the Australian population (mean 50, SD 10).<br>*Interpretation*: This is treated as a continuous variable with higher scores indicate higher energy levels and better mental health.

Sub-module #4: Health problems

Number of items: 17

Description: The Spinal Cord Injury - Secondary Conditions Scale (SCI-SCS) was used to assess the incidence and severity of health problems. Participants were asked to rate how much of a problem they had over the past 3 months with 14 specific secondary health issues, including sleep problems, bowel dysfunction, urinary tract infections, bladder dysfunction, sexual dysfunction, contractures, muscle spasms or spasticity, pressure sores or ulcers, respiratory problems, injuries caused by loss of sensation, circulatory problems, autonomic dysreflexia, postural hypotension and pain. These problems were rated on a five-point scale from 'No problem' to 'Extreme problem'.

In addition, for each problem reported, participants were further asked to indicate whether they had received any treatment, derived from the Self-Administered Comorbidity Questionnaire (SCQ).

To cover additional health problems, a question was added that gives respondents the option of listing up to five additional health problems in a free text format. The free text format was chosen, as health problems vary considerably between persons and countries. Another question related to smoking status was also included.

Adaptation: The original SCI-SCS was developed to measures the effect of 16 health problems on activities and participation, with severity and frequency in one option and difficulties in translation. For Aus-InSCI Community Survey, the modified version of the SCI-SCS was used with 14 questions on a 5-point response options suggested by the Model Disability Survey ranging from 1, "no problem," to 5, "extreme problem".

Interpretation: each variable was treated as a standalone continuous, dichotomous or categorical variable (see details below):

**Variables**	**Data type**
SCI-SCS	• Continuous (sum score ranging from 14 to 70)<br>• Continuous (number of health problems based on severity rating)<br>• Categorial (severity scale)<br>• Dichotomous:<br>(a) no problem (i.e., 1 or 2)<br>(b) problem (3, 4 or 5)
Treatment received	Dichotomous (yes/no)
Other health problems	Categorical
Smoking status	Categorical

Sub-module #5: Activities and participation

Number of items: 18

Description: Severity of activity/participation problems over the last 4 weeks was rated using a five-point scale from no problem to extreme problem for 15 activities including carrying out daily routine, handling stress, doing things that requires use of hands, getting where you want to go, using public transportation, using private transportation, looking after your health, getting

household tasks done, providing care or support to others, interacting with people, intimate relationships and doing things for relaxation or pleasure and mobility activities.

In addition, three questions from Spinal Cord Injury-Functional Index Assistive Technologies (SCI-FI AT) were also included to assess activity limitations and the impact of assistive technology, which were rated on a five-point scale from “able to perform an activity without any difficulty” to “unable to perform an activity”.

Interpretation: The scale was treated as a continuous, dichotomous or categorical variable (see details below):

**Variables**	**Data type**
Activities and participation	• Continuous (sum score ranging from 15 to 75)<br>• Continuous (number of activities based on severity rating)<br>• Categorial (severity scale)
SCI-FI AT	• Continuous (sum score ranging from 3 to 15)<br>• Categorial (severity scale)

NB: The number of reported moderate-extreme problems was calculated with an allowance for a maximum of 2 items to be missed.

Sub-module #6: Independence in activities of daily living

Number of items: 12

Description: A modified version of the self-report version of the Spinal Cord Independence Measure was used to evaluate the independence in activities of daily living. This included 12 questions covering self-care, sphincter management, use of the toilet, and three mobility questions (ability perform a list of five movements unassisted, degree of independence in transferring from bed to a wheelchair, degree of independence in moving moderate distances of 10-100 metres). Participants were asked to evaluate their current status at time of interview.

Adaptation: The original measure includes 17 questions. For Aus-InSCI survey, seven questions were omitted.

Interpretation: An overall summary raw score was calculated (ranging from 0 to 66) and treated as a continuous variable. For the purpose of interpretation, the raw scores were re-scaled to range from 0 (most dependent) to 100 (most independent). In addition, three mobility categories were defined based on degree of independence in moving moderate distances, as below:

- ambulant with or without assistance while walking
- use of a self-operated manual wheelchair, and
- requiring total assistance, using an electric wheelchair or requiring partial assistance to operate a manual wheelchair.

Sub-module #7: Work

Number of items: 16

Description: Six questions were included to assess the work situation including title of job, received vocational rehabilitation, duration of resuming work after SCI, receiving disability pension, current working status and currently engaged in paid work. Additional seven questions were relevant to participant that were employed (current job title, relative working hours and work accessibility and recognition), and three questions in participants who are unemployed (willingly to work and reason for not working).

Adaptation: Questions for this sub-module were drawn from several existing data collection tools including Model Disability Survey, Swiss Spinal Cord Injury Cohort Study Survey and Effort-Reward Imbalance Questionnaire.

Interpretation:

- Pre-injury employment and disability pension were assessed dichotomous data (Yes/No)

	• Vocational service use was measured on a 5-point scale ('a great deal', 'some extent', 'small extent', 'not at all' or 'didn't need vocational rehabilitation services'). The latter two categories for VR services were collapsed given ambiguity in distinction. Visible underemployment (henceforth referred to as underemployment, unless otherwise specified) was assessed using a work hour preference question (more hours, less hours or the same amount).<br>• Main pre- and post-injury job titles were collected as a free text, which were re-coded as categorical data according to the International Standard Classification of Occupations 10 category major classification (ISCO-08) system<br>• Job titles with insufficient details for coding (e.g., 'self-employed') were classed as 'unidentifiable'.
**Sub-module #8: Environmental Factors**	<u>*Number of items*</u>: 14<br><u>*Description*</u>: The Nottwil Environmental Factors Inventory Short Form (NEFI-S) evaluated environmental barriers to participation in society over the past four weeks. A list of fourteen barriers covering issues with accessibility to public places and homes, climatic conditions, attitudes of society, friends, family, colleagues, neighbours, financial difficulties, and the lack of transportation, devices, nursing care, support services, medications, medical aids and supplies, communication devices, or state services were rated either as 'Not applicable', 'No influence', 'Made life a little harder' or 'Made life a lot harder'.<br><u>*Interpretation*</u>: A total summary score was treated as a continuous variable ranging from 0 to 100 where 100 represents the highest possible level of environmental barriers to participation.
**Sub-module #9: Health care services**	<u>*Number of items*</u>: 7<br><u>*Description*</u>: Questions for this sub-module were drawn from Model Disability Survey assessing the quality and accessibility of health service. This included questions about visited health care provider, frequency of hospitalisation, treated respectfully, involvement in decision making for own treatment, unmet health care needs and satisfaction with health.<br><u>*Interpretation*</u>: Each variable was treated as a standalone continuous, dichotomous and/or categorical variable (see details below):

Variables	Data type
Health care provider	Categorical
Hospitalisation	Continuous
Treated respectfully	Categorical
Received information	Categorical
Decision making	Categorical
Health care need	Dichotomous (Yes/No) as well as Categorical
Satisfaction with health services	Categorical

**Sub-module #10: Personal Factors**	<u>*Number of items*</u>: 10<br><u>*Description*</u>: Personal factors were evaluated by asking participants to rate their level of agreement with nine statements addressing self-efficacy (confidence in dealing with opposition and unexpected events, maintaining important contacts and good health, making the big decisions in your life), optimism (has the injury made you stronger, worrying about the future, ability to achieve hopes and dreams) and belonging (feeling included). Responses were on a five-point scale from 'Not at all' to 'Completely'.<br><u>*Adaptation*</u>: The personal factor sub-module consists questions from various existing data collection tools including Global Self Efficacy Scale, Moorong Self Efficacy Scale, Model Disability Survey and WHO QoL questionnaire.

	Interpretation: A summary score for self-efficacy (four questions) was treated as a continuous variable ranging from 4 to 20 rated on a five-point scale from 'Not at all' to 'Completely'. Higher score better outcome.
**Sub-module #11: Quality of life and general health**	*Number of items*: 8<br>*Description*: Quality of life (QoL) was evaluated using 6 questions from the WHO-BREF quality of life instrument. Self-rated overall QoL over the last 14 days was rated on a five-point scale as either very poor, poor, neither, good or very good, while satisfaction with health, activities of daily living, oneself, relationships and living conditions were each rated on a five-point scale from 'Very dissatisfied' to 'Very satisfied'.<br>In addition, two questions from SF-36 were related to general health including health status and health status compared to one year ago.<br>*Interpretation*:<br>• QoL questions were treated as a continuous variable. For the purpose of interpretation, these measures were re-scaled to range from 0 (worst QoL) to 100 (best QoL).<br>• General health questions were treated as categorical variables.
**B. Australian Module**	
Sub-module #1: Access to SCI services	*Number of items*: 6<br>*Description*: This included questions related to Australian state participant were residing, information about rurality, main contact for SCI specific problems as well as overall satisfaction with general practitioner, local general hospital and SCI services.<br>*Interpretation*: These data were used to describe the cohort.
Sub-module #2: Factors impacting functioning	*Number of items*: 23<br>*Description*: This included questions related to pain, description of pain, pain interference with day-to-day activities, overall mood and sleep, shoulder pain, its duration, treatment and treatment effectiveness.<br>This module also included questions related to skin breakdown, its duration, self-management strategies and barries that make skin breakdown difficult to heal.<br>The last section within this module included four question related to fatigue on functioning. Fatigue levels were evaluated from level of agreement with four statements from the Fatigue Severity Scale on how easily and how often the participant experiences fatigue, interference with physical functioning and interference with work, family or social life, rated on a seven-point scale from 'Strongly disagree' to 'Strongly agree'.<br>*Interpretation*: each variable was treated as a standalone continuous, dichotomous and/or categorical variable (see details below):
Sub-module #3:	*Number of items*: 5

**Variables**	**Data type**
Pain interference items	Continuous ranging from 0 to 10
Shoulder pain items	Descriptive as well as categorical
Skin breakdown location	Categorical
Skin breakdown self-strategies	Categorical
Fatigue Severity Scale	Continuous ranging from 4 to 28

Physical Activity	*Description*: The Physical Activity Scale for Individuals with Physical Disabilities (PASIPD) gathers information concerning physical activities performed by the individual for both exercise and other activities. The survey tool instructs participants to recall in the previous seven days how many days per week they engaged in a particular activity (never, seldom (1–2d/wk), sometimes (3–4d/wk), or often (5–7d/wk)), and for how many hours a day they participated in it (<1hr, 1 but <2hr, 2–4hr, >4hr).<br>*Interpretation*: The PASIPD tool multiplies the average hours per day for each item by a metabolic equivalent of task (MET) value associated with the intensity of the activity. It then sums all questions (except first question) to give a score of MET hours per day (hr/day). For this study, only the questions listed under Physical Activity domain were included to provide a MET value of minutes per week as they pertain directly to exercise/physical activity whilst all others pertain to activities of daily living such as garden work, home repairs, occupation, caring for others etc. Of the five questions total, two questions were excluded in the total minute per week scores as the question relates to ambulation specifically not for exercise, whilst all others gather data specifically for exercise.
Sub-module #4: Social Support and Integration	*Number of items*: 10<br>*Description*: Five questions were used to evaluated sense of social support and integration (SI). The first two assessed level of agreement with 'I have little chance to show how capable I am' and 'I feel close to people in my area' on a five-point scale from 'Agree strongly' to 'Disagree strongly'. The remaining three assessed level of agreement with 'Do you feel that people treat you with respect', 'To what extent to you receive help and support from people close to you' and having control over own life on a seven-point scale from 'Not at all' to 'A great deal'.<br>Perceived social injustice (PI) was evaluated based on level of agreement with four statements: 'most people do not understand how severe my condition is', 'I am suffering because of someone else's negligence', 'I just want my life back', and 'it all seems so unfair'. Responses were on a five-point scale from 'Not at all' to 'Completely'.<br>*Interpretation*: A sum score for SI was based on five questions. The first two assessed level of agreement with 'having little chance to show how capable I am' and 'feeling close to people in my area' on a five-point scale from 'Agree strongly' to 'Disagree strongly'. The remaining three assessed level of agreement with 'feeling treated with respect', 'receiving help and support from people close to you' and 'having control over own life' on a seven-point scale from 'Not at all' to 'A great deal'.<br>A sum score for PI was based on level of agreement with four statements, rated on a five-point scale from 'Not at all' to 'Completely'.
Sub-module #5: Sleep Quality	*Number of items*: 24<br>*Description*: Self-reported sleep quality over the past month was measured with the Pittsburgh Sleep Quality Index (PSQI). The instrument contains 10 questions (21 items) asking about sleep habits, problems with sleep, overall sleep quality and impact of sleep on daytime function. The final question asks whether the person has a bed partner or roommate, and if so, how often this person has observed various sleep disturbances. This question is not included in the scoring of the PSQI.<br>The PSQI produces 7 component scores (subjective sleep quality, sleep latency, sleep duration, habitual sleep efficiency, sleep disturbances, use of sleeping medication, and daytime dysfunction) and one global score. Each component score is weighted on a 0–3 interval scale. The global PSQI score is calculated by summing component scores, providing an overall score ranging from 0 to 21. Lower scores correspond to better sleep quality. A score of less than or equal to five is defined as "good sleep", with scores of greater than five defined as "poor sleep". A cut-off score of over 8 may be more sensitive for identifying sleep issues in some populations (i.e., people with TBI and insomnia).

Interpretation: Further analysis of the instrument has led to recommendations for multiple factor scoring methods, rather than a single sleep quality factor (global PSQI score). Two factor and three factor scoring methods have been validated. The three-factor model includes sleep efficiency (combining sleep duration and sleep efficiency component scores), perceived sleep quality (combining subjective sleep quality, sleep latency, and sleep medication component scores), and daily disturbances (combining sleep disturbances and daytime dysfunctions component scores). The two-factor model has been recommended over the three-factor scores. It includes sleep efficiency (combining sleep duration and sleep efficiency component scores) and combines the second and third factors from the three-factor model to provide perceived sleep quality/ daily disturbances (using subjective sleep quality, sleep latency, sleep medication, sleep disturbances and daytime dysfunctions component scores).

Variables	Data type
**PSQI Global Score**	• Continuous (sum score ranging from 0-21)<br>• Categorical (Good sleep <=5; Poor sleep >5)
**PSQI Component Scores**<br>• Subjective sleep quality<br>• Sleep latency<br>• Sleep duration<br>• Habitual sleep efficiency<br>• Sleep disturbances<br>• Use of sleeping medication<br>• Daytime Dysfunction	• Interval (scores ranging from 0-3)
**PSQI Two-factor Score**<br>• Sleep efficiency<br>• Perceived Sleep Quality/ Daily Disturbances	• Interval (sum score ranging from 0-6)<br>• Interval (sum score ranging from 0-15)
