# Supplementary material for: Australian arm of the International Spinal Cord Injury (Aus-InSCI) Community Survey: 2. Understanding the lived experience in people with spinal cord injury
Source: Spinal Cord. 2022 Jun 15;60(12):1069–79. doi: 10.1038/s41393-022-00817-7 (PMC9712098; doi:10.1038/s41393-022-00817-7)
Supplement: Supplementary file 2 — Appendix B: Aus-InSCI National Module [file 41393_2022_817_MOESM2_ESM.pdf]

We thank you very much for participating in the InSCI survey! You have now completed the questions for all countries.

The following questions are specifically for people living in Australia - Please continue or take a break before completing last part of survey.

Access to spinal cord injury services

126. Which state do you live in?
- ○ Australian Capital Territory
- ○ New South Wales
- ○ Northern Territory
- ○ Queensland
- ○ South Australia
- ○ Tasmania
- ○ Victoria
- ○ Western Australia

127. Where do you live?
- ○ Capital city
- ○ Other metropolitan centres (urban centre population > 100,000)
- ○ Large rural centres (urban centre population 25,000-99,999)
- ○ Small rural centres (urban centre population 10,000-24,999)
- ○ Other rural areas (urban centre population < 10,000)
- ○ Remote areas (urban centre population < 5,000)

128. Who is your main contact for spinal cord injury specific problems?
- ○ General practitioner
- ○ Local specialist (e.g. rehabilitation physician, urologist, neurologist)
- ○ Spinal specialist working in a specialist spinal cord injury service/unit
- ○ Others, namely: ✎ ....

129. How satisfied are you with the services provided by your General Practitioner?
- ○ Very satisfied
- ○ Satisfied
- ○ Neither satisfied nor dissatisfied
- ○ Dissatisfied
- ○ Very dissatisfied
- ○ I don't use this service

130. How satisfied are you with the services provided by your local general hospital/s?
- ○ Very satisfied
- ○ Satisfied
- ○ Neither satisfied nor dissatisfied
- ○ Dissatisfied
- ○ Very dissatisfied
- ○ I don't use this service

131. How satisfied are you with the services provided by the Spinal Cord Injury Unit/Service/s in your state?

- ○ Very satisfied
- ○ Satisfied
- ○ Neither satisfied nor dissatisfied
- ○ Dissatisfied
- ○ Very dissatisfied
- ○ I don't use this service

Other factors impacting on functioning

132. Compared to one year ago, how would you rate your function and independence?

- ○ Much better
- ○ Somewhat better
- ○ About the same
- ○ Somewhat worse
- ○ Much worse

133. Have you had any pain during the last seven days including today?

☐ No (go to Question 144)

☐ **Yes** If yes, please answer the following questions about the extent to which pain interferes with your life and how it is being managed:

134. How would you describe your pain?

Check all that apply

- ○ I experience pain that is hot or burning, cold or freezing, pins and needles, tingling, electric shock-like or similar in quality.
- ○ I experience pain that is dull, aching, cramping or tender in muscles in an area or normal sensation.
- ○ The pain only occurs in an area of the body in which I have no feeling on the skin overlying that area.
- ○ The skin over the area of pain is abnormally sensitive to touch and without any surgical scars, ulcers or breaks in the skin.
- ○ The pain is usually unchanged with movement of the painful area.
- ○ The pain is made worse by certain movements, postures or activities.
- ○ I experience pain all the time without any breaks when I am awake (although it may vary in intensity during different times).

135. In general, how much has pain interfered with your day-to-day activities in the last week?

No Interference ←→ *Extreme interference*

○	○	○	○	○	○	○	○	○	○	○
0	1	2	3	4	5	6	7	8	9	10

136. In general, how much has pain interfered with your overall mood in the last week?

No Interference ←→ *Extreme interference*

○	○	○	○	○	○	○	○	○	○	○
0	1	2	3	4	5	6	7	8	9	10

137. In general, how much has pain interfered with your ability to get a good night's sleep in the last week?

No Interference ←→ *Extreme interference*

○ 0 ○ 1 ○ 2 ○ 3 ○ 4 ○ 5 ○ 6 ○ 7 ○ 8 ○ 9 ○ 10

138. During the last 3 months, have you experienced shoulder pain lasting for more than one day?

Shoulder pain is defined as pain in or around the shoulder area.

- ○ No (please go to Question 141)
- ○ Yes, I have experienced pain in the right shoulder
- ○ Yes, I have experienced pain in the left shoulder
- ○ Yes, I have experienced pain in both shoulders

139. For how long have you experienced shoulder pain?

- ○ Less than 3 months
- ○ 3 – 12 months
- ○ 1 – 5 years
- ○ 6 – 10 years
- ○ 11 – 15 years
- ○ More than 15 years

140. Over the last 3 months, has your shoulder pain prevented you from doing things in your everyday life?

- ○ No
- ○ Yes, please specify: ……………………………………………………………………..

141. Which of these treatments/strategies do you use to help manage your pain – *select all that apply*?

Check all that apply

- ☐ Take over the counter medicines (e.g. Paracetamol, Ibuprofen)
- ☐ Take prescribed nerve pain medications (e.g. Lyrica, Neurontin)
- ☐ Take prescribed opioid medications (e.g. Endone, Oxycodone, Fentanyl patch)
- ☐ Take non-prescribed medications (e.g. marijuana)
- ☐ Use alcohol to dull the pain
- ☐ Attend physiotherapy
- ☐ Seek emotional or psychological support
- ☐ Attend peer support groups
- ☐ Keep physically active (e.g. walking, fitness programs, daily chores)
- ☐ Try to get enough sleep
- ☐ Practice relaxation / meditation (e.g. mindfulness) techniques regularly
- ☐ Try to eat a healthy diet and keep to a healthy weight
- ☐ Attend manual therapies (e.g. massage, acupuncture, osteopathy, chiropractic)
- ☐ Receive other complementary therapies or treatments (e.g. homeopathy, naturopathy, Chinese herbalism)
- ☐ Others, namely: ………………………………………………………………..

142. Please rate how effective you find the treatments you use for managing your pain?

Ineffective ←→ *Extremely effective*

○ 0 ○ 1 ○ 2 ○ 3 ○ 4 ○ 5 ○ 6 ○ 7 ○ 8 ○ 9 ○ 10

143. **Where do you receive advice and help to manage your pain – *select all that apply*?**

Check all that apply

- ☐ General Practitioner
- ☐ Spinal Medical Specialist
- ☐ Pain Specialist
- ☐ Physical therapist (eg: physiotherapist, exercise physiologist, occupational therapist)
- ☐ Psychologist / Counsellor
- ☐ Hospital (including Emergency Department)
- ☐ Chronic Pain Clinic (which provides multi-disciplinary care)
- ☐ Online Pain Management Courses (e.g. e-centre clinic)
- ☐ Complementary therapist (e.g. acupuncturist, homeopath, naturopath, Chinese herbalist)
- ☐ Website (e.g. ACI Pain Management Network - SCI Pain pages)
- ☐ Other, please specify: ……………………………………………………………………………

The next four questions ask about the impact of fatigue on your functioning. *Please choose a number for each statement from 1 to 7 that indicates your level of agreement with each statement, where 1 indicates "Strongly disagree" and 7 indicates "Strongly agree"*

**Statement**	*1 Strongly disagree*	*2 Disagree*	*3 Slightly disagree*	*4 Neither agree nor disagree*	*5 Slightly agree*	*6 Agree*	*7 Strongly agree*
**144. I am easily fatigued.**	○	○	○	○	○	○	○
**145. Fatigue interferes with my physical functioning.**	○	○	○	○	○	○	○
**146. Fatigue causes me frequent problems.**	○	○	○	○	○	○	○
**147. Fatigue interferes with my work, family or social life.**	○	○	○	○	○	○	○

The next few questions ask about problems with skin breakdown that may affect your health, function and participation.

148. **In the last 12 months, have you had an area of skin breakdown / ulcer / pressure injury?**

- ○ No (please go to Question 153)
- ○ Yes, I have experienced one pressure injury / ulcer
- ○ Yes, I have experienced two or more pressure injuries / ulcers
- ○ Yes, I have experienced skin breakdown, but it was not caused by pressure (eg. burn)
- ○ Other, please specify: ……………………………………………………………………………

If Yes, please indicate the location of pressure ulcer/s over last 12 months:

Please fill in the following diagram ***with one tick for each ulcer***:

	Right	Mid-line	Left
Occiput (back of head)			
Scapula (shoulder blade)			
Elbow			
Ribs			
Spinous processes (bony prominences over vertebra)			
Sacrum (triangular bone at base of spine) & Coccyx (tail bone)			
Ischial tuberosity (buttock, where we sit)			
Trochanter (area over hip joint when lying on side)			
Genitals			
Knee			
Ankle over malleolus (bony prominence)			
Heel			
Other location			

149. How long did the area of skin breakdown / ulcer / pressure injury take to heal completely?

○ Less than 1 week
○ 1-4 weeks
○ 1-3 months
○ 3-6months
○ 6-12 months
○ More than 12 months

150. Did the skin breakdown / ulcer / pressure injury require an admission/s to hospital?

○ No
○ Yes, I spent ………..days in hospital

If Yes, was the area of skin breakdown / ulcer treated surgically

○ No
○ Yes

151. Were you referred to a Spinal Plastics Service / Spinal Pressure Care Clinic based in your capital city?

○ No
○ Yes

152. Do you have a skin breakdown / ulcer / pressure injury that has never completely healed or that breaks down frequently?

○ No
○ Yes

153. What do you do when you discover you have a red mark, skin breakdown, ulcer or pressure injury on an area you sit on?

Check all that apply

☐ Maintain complete 24 hour per day lying in bed (I do **not** get up to go to the toilet or have a shower)
☐ Stay in bed (but I do get up to go to the toilet and have a shower)
☐ Reduce time sitting

- ☐ Seek advice from an Occupational Therapist or Seating Therapist
- ☐ Contact local medical or nursing services
- ☐ Contact the Spinal Unit or Spinal Plastics Service / Spinal Pressure Care Clinic for advice
- ☐ I am able to increase the amount of care I receive
- ☐ I am able to upgrade my equipment if required (e.g. mattress, wheelchair cushion, commode)
- ☐ Other, please specify: ✎ ……………………………………………………………………

154. Please name any barriers (up to five) that make it difficult to heal a skin breakdown, ulcer or pressure injury:

☐ No barriers experienced

✎ ………………………………………………………………………

✎ ………………………………………………………………………

✎ ………………………………………………………………………

✎ ………………………………………………………………………

✎ ………………………………………………………………………

Physical Activity

This section is about your current level of physical activity and exercise. Please remember there are no right or wrong answers. We simply need to assess your current level of activity.

155. During the past 7 days, how many days did you *walk, wheel, (hand) cycle outside* your home *other than specifically for exercise*. For example, getting to work or class, walking the dog, shopping, or other errands?

_______ days per week [score options: 0 – 7] *If 0 days → Go to question 156.*

On average, how many minutes per day did you spend walking, wheeling or (hand) cycling outside your home?

_______ minutes per day.

156. During the past 7 days, how many days did you engage in *light sport or recreational activities* such as bowling, golf with a cart, hunting or fishing, darts, billiards or pool, therapeutic exercise (physical or occupational therapy, stretching, use of a standing frame) or other similar activities?
Note: Light sport or recreational activities require very light physical effort; these activities make you feel like you are working a little bit, but you can keep doing them for a long time without getting tired.

_______ days per week [score options: 0 – 7] *If 0 days → Go to question 157.*

On average, how many minutes per day did you spend in these *light sport or recreational* activities?

_______ minutes per day.

157. During the past 7 days, how many days did you engage in moderate sport and recreational activities such as doubles tennis, softball, golf without a cart, ballroom dancing, wheeling or pushing for pleasure or other similar activities?
Note: Moderate sport and recreational activities require some physical effort; these activities make you feel like you are working somewhat hard, but you can keep doing them for a while without getting tired.

_______ days per week [score options: 0 – 7] *If 0 days → Go to question 158.*

On average, how many minutes per day did you spend in these moderate sport and recreational activities?

_______ minutes per day.

158. During the past 7 days, how many days did you engage in *strenuous sport and recreational* activities such as jogging, wheelchair racing (training), off-road pushing, swimming, aerobic dance, arm cranking, cycling (hand or leg), singles tennis, rugby, basketball, walking with crutches and braces, or other similar activities?
Note: Strenuous sport and recreational activities require a lot of physical effort; these activities make you feel like you are working really hard, almost at your maximum. You cannot do these activities for very long without getting tired. These activities may be exhausting.

_______ days per week [score options: 0 – 7] *If 0 days → Go to question 159.*

On average, how many minutes per day did you spend in these strenuous sport or recreational activities?

_______ minutes per day.

159. During the past 7 days, how many days did you do any exercise specifically to increase muscle strength and endurance, such as lifting weights, push-ups, pull-ups, dips, or wheelchair push-ups, etc?

_______ days per week [score options: 0 – 7] *If 0 days → Go to question 160.*

On average, how many minutes per day did you spend in these *exercises to increase muscle strength and endurance*?

_______ minutes per day.

Society / Social integration

160. In my daily life I get very little chance to show how capable I am.

- ○ Agree strongly
- ○ Agree
- ○ Neither agree nor disagree
- ○ Disagree
- ○ Strongly disagree

161. I feel close to the people in my local area.

- ○ Agree strongly
- ○ Agree
- ○ Neither agree nor disagree
- ○ Disagree
- ○ Strongly disagree

162. When I hit a major problem at work or in attempting to return to work, I have co-operative co-workers or supervisors that help me overcome it.

- ○ Agree strongly
- ○ Agree
- ○ Neither agree nor disagree
- ○ Disagree
- ○ Strongly disagree

Please choose a number for each statement from 0 to 6 that indicates your level of agreement with each statement, where 0 indicates "Not at all" and 7 indicates "Strongly agree"

		Not at all 0	1	2	3	4	5	*A great deal* 6
**163.**	**Do you feel that people treat you with respect?**	○	○	○	○	○	○	○

		Not at all 0	1	2	3	4	5	*Completely* 6
**164.**	**To what extent do you receive help and support from people you are close to when you need it?**	○	○	○	○	○	○	○

		No control 0	1	2	3	4	5	*Extreme control* 6
**165.**	**During the past week how much control do you feel that you have had over your life?**	○	○	○	○	○	○	○

When a traumatic injury or disease causes sudden spinal cord impairment, it can have profound effects on our lives. The following questions are from a scale designed to assess how your injury has affected your life. Listed below are four statements describing different thoughts and feelings that you may experience when you think about your injury.

Please indicate the degree to which you have these thoughts and feelings when you think about your injury on a 5-point scale with the endpoints where 0 indicates "not at all" and 4 indicates "all the time".

	**Statement**	*Not at all* 0	1	2	3	*All the time* 4
**166.**	**Most people don't understand how severe my condition is.**	○	○	○	○	○
**167.**	**I am suffering because of someone else's negligence.**	○	○	○	○	○
**168.**	**I just want my life back.**	○	○	○	○	○
**169.**	**It all seems so unfair.**	○	○	○	○	○

Sleep Quality

People with a spinal cord injury commonly report problems with sleep. This final series of questions relate to your usual sleep habits during the past month only. Your answers should indicate the most accurate reply for the majority of days and nights in the past month. **Please answer all questions.**

170. During the past month, what time have you usually gone to bed at night? ✐.................................

171. During the past month, how long (in minutes) has it usually taken you to fall asleep each night?
✐.................................

172. During the past month, what time have you usually gotten up in the morning? ✐.................................

173. During the past month, how many hours of actual sleep did you get at night? (This may be different than the number of hours you spent in bed.) ✐.................................

	During the past month, how often have you had trouble sleeping because you…	*Not during the past month*	*Less than once a week*	*Once or twice a week*	*Three or more times a week*
174.	Cannot get to sleep within 30 minutes	○	○	○	○
175.	Wake up in the middle of the night or early morning	○	○	○	○
176.	Have to get up to use the bathroom	○	○	○	○
177.	Cannot breathe comfortably	○	○	○	○
178.	Cough or snore loudly	○	○	○	○
179.	Feel too cold	○	○	○	○
180.	Feel too hot	○	○	○	○
181.	Have bad dreams	○	○	○	○
182.	Have pain	○	○	○	○
183.	Other reason(s), please describe: ✐...	○	○	○	○
184.	During the past month, how often have you taken medicine to help you sleep (prescribed or “over the counter”)?	○	○	○	○
185.	During the past month, how often have you had trouble staying awake while driving, eating meals, or engaging in social activity?	○	○	○	○

186. During the past month, how much of a problem has it been for you to keep up enough enthusiasm to get things done?

- ○ No problem at all
- ○ Only a very slight problem
- ○ Somewhat of a problem
- ○ A very big problem

187. **During the past month, how would you rate your sleep quality overall?**

- ○ Very good
- ○ Fairly good
- ○ Fairly bad
- ○ Very bad

		No bed partner or room mate	*Partner / room mate in other room*	*Partner in same room but not same bed*	*Partner in same bed*
188.	**Do you have a bed partner or room mate?**	○	○	○	○

	**If you have a room mate or bed partner, ask him/her how often in the past month you have had:**	*Not during the past month*	*Less than once a week*	*Once or twice a week*	*Three or more times a week*
189.	**Loud snoring**	○	○	○	○
190.	**Long pauses between breaths while asleep**	○	○	○	○
191.	**Legs twitching or jerking while you sleep**	○	○	○	○
192.	**Episodes of disorientation or confusion during sleep**	○	○	○	○
193.	**Other restlessness while you sleep, please describe:** ✎	○	○	○	○

Once again we thank you very much for participating in this survey! You have now completed ALL questions.
